# Combination of Freezing, Low Sodium Brine, and Cold Smoking on the Quality and Shelf-Life of Sea Bass (*Dicentrarchus labrax* L.) Fillets as a Strategy to Innovate the Market of Aquaculture Products

**DOI:** 10.3390/ani11010185

**Published:** 2021-01-14

**Authors:** Concetta Maria Messina, Rosaria Arena, Giovanna Ficano, Laura La Barbera, Maria Morghese, Andrea Santulli

**Affiliations:** 1 Dipartimento di Scienze della Terra e del Mare DiSTeM, Laboratorio di Biochimica Marina ed Ecotossicologia, Università degli Studi di Palermo, Via G. Barlotta 4, 91100 Trapani, Italy; rosaria.arena@unipa.it (R.A.); giovanna.ficano@unipa.it (G.F.); andrea.santulli@unipa.it (A.S.); 2 Istituto di Biologia Marina, Consorzio Universitario della Provincia di Trapani, Via G. Barlotta 4, 91100 Trapani, Italy; labarbera@consunitp.it (L.L.B.); maria.morghese@unipa.it (M.M.)

**Keywords:** *Dicentrachus labrax*, aquaculture, cold smoking, sodium replacement, shelf-life, fillets, low-salt product, fish quality

## Abstract

**Simple Summary:**

The growing fish consumption driven by the increased production, the concomitant reduction in wastage, and the huge amount of fish food traded globally, makes it important to address the sustainability, profitability, security, and safety issues related to the seafood production sector. Toward this direction, innovative methods extending shelf-life, maintaining seafood quality, safety and nutritional characteristics and that open new market opportunities, satisfy consumer preferences, and improve product traceability are required. Answering this call, this study aimed to develop a new value added product exploiting a species largely cultured in Italy (i.e., European sea bass). In particular, sea bass fillets were cold smoked using both fresh and frozen fillets to understand the effectiveness of this kind of processing on developing a new fish product and whether the quality of the raw material that could be affected by freezing and thawing could change the quality of the final product. It was seen that the quality of the raw material was affected by the time of frozen storage and that these starting conditions consequently impaired the quality of the smoked fillets. However, cold smoking was proven to be an effective process to develop a valuable product contributing to the growth of the aquaculture sector.

**Abstract:**

Aquaculture is playing a leading role in both meeting the growing demand for seafood and increasing the sustainability of the fish production sector. Thus, innovative technologies that improve its sustainability, competitiveness, and safety are necessary for growth in the sector. This study aimed to develop cold smoked sea bass fillets from aquaculture. The aptitude of frozen and fresh fillets for cold smoking was investigated by processing both fresh and thawed fillets kept previously at −20 °C for 15, 30, 60, and 90 days. Moreover, to develop a low-salt product, fillets were immersed in low-sodium or standard brine. Sensory, biochemical, and physical-chemical analyses were performed on both the raw fillets and the smoked fillets during vacuum packaged storage for 35 days at 1 ± 0.5 °C. Young modulus values, representative of texture and sensory evaluation, showed that the quality of fresh fillets was better compared to the thawed ones, thus affecting the quality of the final product as the correlation between parameters showed (principal component analysis). Cold smoking was effective in both maintaining the total volatile basic nitrogen (TVB-N) below the threshold for spoilage and preventing lipid peroxidation. Moreover, partial sodium replacement by potassium did not alter the sensory attributes of smoked fillets, which maintained high scores up to 21 days.

## 1. Introduction

Fish has always been considered an important part of human consumption for both its good taste and the high nutritional value of its flesh. According to the FAO [1], aquaculture as a food production system has largely grown over the last decades due to the increasing demand for seafood, function of the growing world population, and the increased awareness of the beneficial effects on human health related to fish consumption. Among the most exploited species in the aquaculture sector is European sea bass (*Dicentrarchus labrax*) [2], which is widely cultured in Mediterranean areas, with Greece, Turkey, Italy, Spain, Croatia, and Egypt the biggest producers [1]. Specifically, the Italian production, which can rely on good nutritional and safety standards [3,4], has to satisfy the general need to enlarge production with innovative products. To do so, it is important to assess all parameters previously related to the quality evolution during shelf-life in order to support the production and contribute to the growth of the sector.

Indeed, given that growing fish consumption has been driven by the increased production, the concomitant reduction in wastage, and the huge amount of fish food traded globally [1], important issues have to be addressed with regard to the environmental and economic sustainability, profitability, security, and safety of the seafood production sector. Toward this direction, innovative methods that extend shelf-life, maintain seafood quality, safety, and its own nutritional characteristics and that open new market opportunities, satisfy consumer preferences, and improve product traceability are required. 

Cold smoking is among the techniques widely used for adding value to seafood products as well as to extend their shelf-life, maintaining high quality and sensory features of the product during storage [5,6]. The effectiveness of the method is due to both smoke components such as phenols, aldehydes, ketones, hydrocarbons, esters, ethers, alcohols and the drying and salting phases that lower water activity, a parameter that influences microbial growth. Moreover, chloride ions are toxic for some microorganisms, inhibiting enzymatic systems [7]. In addition, the antioxidant activity of smoke components prevents healthy biochemical compounds like omega-3 polyunsaturated fatty-acids to be oxidized, for instance, eicosapentaenoic acid (EPA) and docosahexaenoic acid (DHA), which exert a strong positive influence on human health, for example, by playing a significant role in preventing cardiovascular and cognitive diseases and reducing the symptoms in rheumatoid arthritis [8]. It is also important to consider the synergistic effect of cold smoking with appropriate storage conditions (i.e., preservation of the processed product under vacuum-packaging or modified atmosphere packaging on extending the shelf-life of fish products by retarding microbial growth). Combination of these techniques with natural antioxidants was proven to extend dolphinfish shelf-life and prevent the loss of high beneficial compounds like EPA and DHA [9,10,11].

Several studies have reported the effectiveness of cold smoking on salmon (*Salmo salar*) by dealing with several technological aspects related to the technique and the quality of the raw material in order to improve this technique, which is able to prolong fish shelf-life and produce a value-added seafood product [12,13,14,15,16]. Moreover, it has been used over the years in many other fish species in order to make available all year round seasonal species as in the case of dolphinfish (*Coryphaena hippurus*) [6,9,17], but even to enhance fishery species with excess catches such as sardines (*Sardina pilchardus*) [6,17] and herring (*Clupea harengus*) [18].

To deal with fluctuations in cold-smoked salmon demand [16], the aptitude of fresh and frozen salmon fillets for cold smoking has been investigated by several authors [13,14,16,19]. For this purpose, several quality features need to be studied because the frozen storage may affect physicochemical and sensory aspects of the processed product. Sigurgisladottir et al. [13] studied the effect of freezing and thawing of the raw material on the microstructure and texture of the smoked product where it was seen that these previous conditions determined the muscle fiber shrinkage and the increase of the extracellular space, which could determine the liquid leakage of the smoked product. Nevertheless, a reduction in the yield during the smoking process was not observed. Freezing salmon fillets before smoking and keeping them frozen for up to 24 h before consumption increased the shelf-life of the smoked product compared to refrigerated smoked fillets and improved several flesh properties such as color intensity and firmness, which can make the product more acceptable to consumers [19]. Another study showed that the characteristics of raw salmon for cold smoking affected the smoked product in terms of yield and sensory features. Indeed, smaller, leaner individuals appeared to be more sensitive to freezing before smoking [20].

The aptitude of frozen fish for cold-smoking was investigated in other fish species, for instance, to make the smoked product available all year round [17]. It was seen that both sardines and dolphinfish fillets could be frozen for up to two/four and twelve months, respectively, to gain high quality smoked products as expressed by biochemical parameters, protein functionality, and sensory scores. It was also noticed that few studies had dealt with the effect of freezing/thawing of the fish muscle on the quality of the smoked product.

Salt is commonly used for fish processing as well as in the cold smoking process because it helps to increase shelf-life. Salt has an important effect on water retention capacity (WHC), fat bonding, color, taste, and texture [21]. However, high salt (sodium) intake is associated with raised blood pressure, hypertension, and cardiovascular diseases, the first cause of death and disability in adults worldwide. Clinical guidelines do not treat the majority of individuals with the tendency of high blood pressure with drugs, therefore, a population-approach through non pharmacological measures (diet and life-style) is the most feasible option, as recommended by the World Health Organization (WHO) [22] and adopted under a UN Resolution of the 66th World Health Assembly in 2013 [23].

High salt intake, as above-mentioned, is associated with high blood pressure and a moderate reduction in salt consumption can cause a significant reduction in blood pressure [24] and reduced cardiovascular events. The possibility of creating products with low sodium content could generate new value-added functional foods for new markets. 

Salt substitutes with similar properties and whose consumption is not deleterious for humans have also been considered in fish products [25]. Potassium chloride (KCl) is the most popular inorganic salt replacer, but is characterized by acrid, metallic, and bitter tastes. Moreover, considering that sodium chloride (NaCl) substitution may lead to changes in several physical-chemical parameters, it is important to investigate how NaCl replacement could affect the final product quality and which percentage is the best one to consider in the process, also taking into account that the moisture content and the water activity need to be very similar to the traditional product [26]. Fuentes et al. [21,26,27] investigated the effect of partial sodium replacement with potassium on European sea bass smoked with liquid smoke solutions in order to develop a healthier product. It was seen that a substitution percent higher than 50% negatively affected the taste of the smoked product [26], inferring the sensory acceptability of the smoked product. Moreover, it was seen that the type of vacuum and modified atmosphere packaging increased the shelf-life of smoked sea bass compared to air, highlighting the importance of using appropriate storage conditions to prevent product spoilage and extend the shelf-life [27]. Moreover, the feasibility of partial replacement of NaCl with KCl has been investigated on other fish species such as salmon and herring [28,29]. It has been shown that in smoked herring, the best substitution percent of NaCl with KCl was not higher than 40% [29].

In order to develop a new value-added product by using the traditional aquaculture species *D. labrax*, thus contributing to the diversification of seafood products and satisfying the consumer’s preferences and sustainability needs, the aim of the current study was to develop cold smoked sea bass fillets by replacing sodium chloride with potassium chloride in the salting phase. In particular, the fillets were cold smoked by using either a NaCl brine solution or a NaCl/KCl (1:1 *w/w*) brine solution in order to provide evidence on the effectiveness of the partial sodium replacement with potassium on the quality of the cold smoked product. In addition, the present study provides evidence to the production sector on the suitability of both frozen and fresh sea bass fillets for cold smoking by applying the process to both fresh and thawed fillets.

## 2. Materials and Methods 

### 2.1. Fish Sampling and Processing

A total of 195 fillets of farmed sea bass (*D. labrax*) were sampled immediately after production at the processing sector of the aquaculture facility located in Sicily (Italy), stored in ice, and brought to the laboratory under cold storage. The fillets were divided into five lots, each one with 39 fillets.

**Figure 1:**
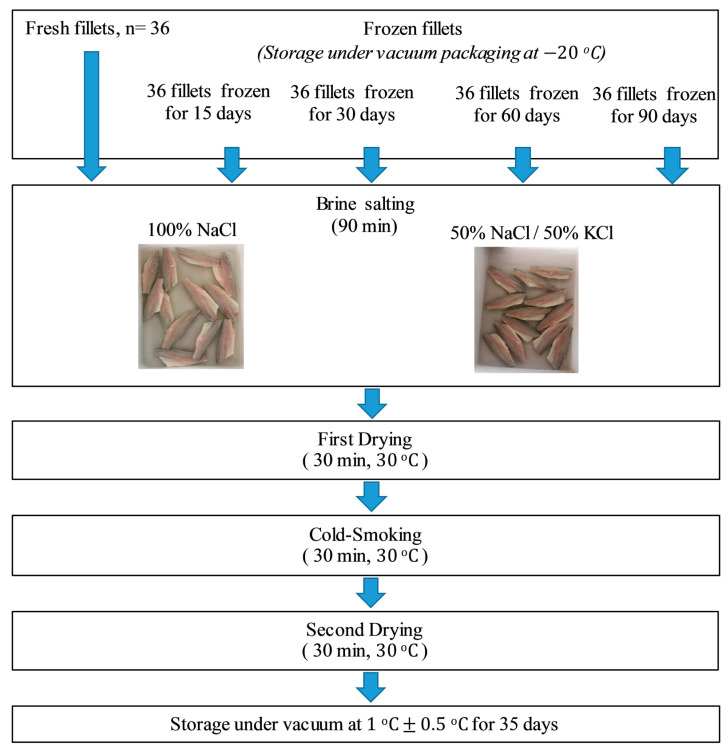
Flowchart of the experimental design.

The first batch of 39 fillets was immediately used for the smoking process, in particular, three fresh fillets of this lot were used for the analysis at T0 (i.e., the time right before processing), while the remaining fresh fillets of this lot were immediately smoked (*N* = 36), as shown in Figure 1.

The other four batches (each of 39 fillets) were stored under vacuum in Foodsaver (HDPE) and nylon bags (http://www.gopack.it), subjected to rapid freezing at −80 °C, and maintained at −20 °C for 15, 30, 60, and 90 days before processing. As far as the frozen fillets were concerned, they were placed in air-permeable low-density polyethylene (LDPE) bags and thawed at 4 °C for 8 h before processing.

### 2.2. Salting, Smoking, Storage, and Sampling

The whole process was represented by four steps: brine salting, first drying, cold smoking, and second drying (Figure 1).

For all batches, 18 fillets were immersed in standard brine (STD) composed of 15% (*w/v*) NaCl solution, and the remaining 18 fillets of the same batch were immersed in a 15% (*w/v*) NaCl/KCl solution (1:1 *w/w*). For both treatments, a fillet:brine ratio equal to 1:4 was used [9,30]. The brine salting lasted 90 min, after which, the samples were dried for 30 min at a temperature that never exceeded 30 °C. Then, all treatments were cold smoked for the same time and at the same temperature as described in Messina et al. [9]. Briefly, cold smoking was performed using a Moduline oven model FA082E (Scubla srl, Remanzacco (Ud), Italy) for 30 min at 30 °C by employing a mixture of the hacks pane (*Fagus* spp.) (Scubla srl, Remanzacco (Ud) Italy) and smoke shavings and Segamehl smoked flour (Scubla srl, Remanzacco (Ud) Italy).

As a final step, the experimental fillets were dried again for 30 min at 30 °C. At the end of the process, fillets were packaged under vacuum and stored at 1 ± 0.5 °C for 35 days. Samples were analyzed at regular intervals defined previously (1, 7, 14, 21, 28, and 35 days after smoking), during which they were monitored through a multidisciplinary approach (i.e., by biochemical, sensory and physical-chemical analyses).

Each lot of fillets was treated as described above in order to carry out five tests: the first smoking (I) was carried out by treating fresh fillets; in the second (II), third (III), fourth (IV), and fifth (V) smoking, fillets kept frozen for 15, 30, 60 and 90 days, respectively, were treated.

### 2.3. Physical-Chemical Parameters

#### 2.3.1. Color

The analysis was carried out using a Konica Minolta colorimeter (Osaka, Japan), and the results were reported according to the CIE system [31]. Lightness (L*), redness index (a*), yellowness index (b*), Hue, and Chroma were recorded, whereas the numerical total color difference (ΔE) between samples was calculated by Equation (1):

(1) $$\Delta E^{*}=\sqrt{{\left({\Delta L}^{*}\right)}^{2}+{\left({\Delta a}^{*}\right)}^{2}+{\left({\Delta b}^{*}\right)}^{2}}$$

where ΔL*, Δa*, and Δb* are the differentials among the color parameters of the samples over the course of shelf-life and the color parameters of the samples at T0.

To perform this kind of analysis, first, the instrument was calibrated, and the instrument reading head was placed on the surface of the fillet. The color evaluation was performed on the fillet in two dorsal regions along the cephalo-caudal direction. The analysis was performed in triplicate. With regard to the kind of observer, it was 2° while the source of light was D65.

#### 2.3.2. Texture Profile Analysis

Two small fragments (1.8 cm Ø), obtained from the same portions of each fillet, were used. The analysis was performed at room temperature using an Instron Texture Analyzer Mod. 3342 (Turin, Italy).

The measured parameters were Hardness (*N*) and the Young modulus or modulus of deformability (*N*/mm^2^) (i.e., the force and the slope of the curve at 50% compression, respectively) [9,32]. The analysis was performed in triplicate. In particular, for each replicate as above-mentioned, two fragments per fillet along the dorsal margin were considered. The samples were maintained in ice before the analysis. 

#### 2.3.3. Water Holding Capacity

Water holding capacity was determined using the method described by Teixeira et al. [33] with some modifications. Briefly, chopped muscle (2 g; *Ws*) was placed in a centrifuge tube along with filter paper (also weighted, *Wi*) and centrifuged at 3000 g for 10 min at 20 °C. After centrifugation, the sample was removed and the filter paper weighted (*Wf*). The moisture in the samples was determined according with the AOAC method [34]. WHC was calculated following Equation (2) and expressed as gram of water in the sample after centrifugation per 100 g of water initially present in the sample. The analysis was performed in triplicate.

(2) $$WHC=\frac{Ws*\frac{Moisture\%}{100}-\left(Wf-Wi\right)}{Ws*\frac{Moisture\%}{100}}$$

#### 2.3.4. Muscular pH

The muscular pH of the fillet was measured at three points along the lateral line with a Crison pH meter (Barcelona, Spain) equipped with a BlueLine pH 21 Schott Instruments (Weilheim, Germany) combined electrode.

#### 2.3.5. Determination of the NA, K, and Salt Content

Na, K, and salt were determined according to the technique described by Greiff et al. [35] with modification. Briefly, about 2 g of sample were homogenized in distilled water (1:4 *w/v*) using an Ultraturrax T25 (IKA, Labortechnik, Staufen, Germany) and centrifuged at 5000 g for 5 min. The extract was filtered on cotton wool. Na and K content was directly measured with a Horiba B-722 (Na^+^) and B-731 (K^+^) LAQUAtwin Compact Ion Meter (Horiba Instruments, Inc., Kyoto, Japan). A conductivity meter (Model 131 Analytical Control, Milano, Italy) was used to measure the salinity. The obtained value expressed in µS cm^−1^ was converted into salinity (expressed in ‰). The values obtained were expressed in g/100 g of product. The tests were carried out on the newly processed product. All analyses were performed in triplicate. 

#### 2.3.6. Water Activity Determination

Water activity (aw) was measured with a fast water activity meter (HP23-AW Rotronic, AG, Bassersdorf, Switzerland). The temperature at which the water activity was measured was equal to 21 °C. The analysis was performed in triplicate.

### 2.4. Biochemical Parameters Related to the Shelf-Life

After the physical-chemical and sensory analyses, in order to carry out further analysis, the fillets were cold homogenized. 

The total lipids (TL) were determined according to Folch, Lees, and Stanley [36] and the fatty acid (FA) methyl esters were determined by the method of Lepage and Roy [37]; gas-chromatography was carried out following the operating conditions described in Messina et al. [38].

The production of thiobarbituric acid reactive substances (TBARS) was determined using the method described by Botsoglou et al. [39]. The total volatile basic nitrogen (TVB-N) was measured by direct distillation of the homogenized samples according to the EU Commission Decision 95/149/EC [40]. 

All analyses were performed in triplicate.

### 2.5. Sensory Analysis

Sensory analysis of raw fillets with skin was conducted by a panel of six trained judges in accordance with an adapted version of the EU scheme [41,42]. The analysis consisted of the evaluation of skin odor and flesh texture, brightness, and color, as illustrated in Table 1. The numeric scores 3, 2, 1 and lower than 0.5 were assigned, respectively, to the E class (very fresh, extra quality), A class (fresh, good quality), B class (bad quality but still fit for sale), and unfit for sale, in order to obtain a quantitative evaluation of parameters [42]. The analysis was performed in triplicate.

As far as the smoked fillets were concerned, the sensory assessments were performed on fillets by a six-member panel according to the protocol developed by Fuentes et al. [26] in which attributes such as odor, color, appearance, taste were assessed by using a 5-point descriptive scale (1 = very unpleasant; 2 = unpleasant; 3 = neutral; 4 = less typical; 5 = very typical). The analysis was performed in triplicate.

### 2.6. Statistical Analysis

Statistical differences were evaluated for each parameter with analysis of variance (ANOVA). The differences among the mean values were assessed using the Student–Newman–Keuls test. The degree of heterogeneity was measured by the Cochran test [43]. Principal component analysis was performed including eight variables on an individual basis. Data were processed for one-way ANOVA and principal component analysis (PCA) by Statistica (version 8.0, Statsoft, Inc., Tulsa OK, USA).

## 3. Results and Discussion

### 3.1. Color

Color is one of the main qualitative aspects of interest to the consumer before purchase [27]. CIE L* a* b*, hue (h*), chrome (C*) and ΔE appear in the Supplementary Materials (Supplementary Table S1 and Table S2). Regarding the L* parameter, differences were observed between the thawed fillets compared to the fresh fillets (Supplementary Table S1). These findings agreed with those obtained in Atlantic salmon fillets [14,44] in which L* increased significantly with frozen storage, showing that this parameter is affected by freezing. 

This study showed that L* in smoking I remained stable during the storage time for both samples with significant increase (*p* < 0.05) at T35 (see Supplementary Table S1 in the Supplementary Materials). This trend was observed in all subsequent smoking processes. Birkeland et al. [12] observed a reduction in lightness after cold smoking of salmon fillets; the lightness was more reduced in dry-salted than injection-salted fillets, highlighting that the difference could be due to a difference in the water content. Moreover, an increase in lightness and reduction in redness was associated with a loss of quality in cold smoked salmon influenced by the duration of the brine salting treatment [15]. Yagiz et al. [45] treated *C. hippurus* fillets with high pressure (HP) and reported that the L* value increased slightly as a function of increased pressure and general loss of quality.

A positive a* value indicates redness. As far as this parameter was concerned, a constant trend for smoking I was observed, where no significant differences (*p* > 0.05) were observed between the two treatments (Supplementary Table S1), highlighting the preservative effect of smoke on lipid oxidation as shown by other authors [9]. From smoking II, the a* value tended to decrease compared to the thawed fillets (Supplementary Table S1). This difference was significant (*p* < 0.05) after seven days of storage in all smoking processes. 

The same pattern related to the increase of L* (lightness) in the thawed fillets compared to fresh fillets was observed for b* (yellowness), which was significantly different (*p* < 0.05) between the fresh fillets and frozen fillets after thawing, which tended to increase in the latter ones (Supplementary Table S1). The same results were obtained in Atlantic salmon fillets, which showed an increase in yellowness due to freezing [14,44]. 

In all smoking processes, the b* value decreased significantly after processing (*p* < 0.05), which then increased during storage (Supplementary Table S1). This increase was more significant (*p* < 0.05) in smoking V, where b* values of 6.48 ± 2.17 and 4.89 ± 1.29 for NaCl and NaCl/KCl samples, respectively, were observed at T35. This pattern may be indicative of an increased yellowness of fish muscle as a result of the browning of the bloodline due to heme protein oxidation and a corresponding reduction in a* [46]. No significant differences (*p* > 0.05) were observed between the two treatments.

The smoking process caused significant variation to the color parameters (*p* < 0.05) and the effect on the color variation after smoking was well observable from the values shown in the ΔE (Supplementary Table S2). The first smoking, performed on fresh samples, was the one that showed a lower ΔE for both treatments, a value that remained constant during the shelf-life. Samples that were frozen and subsequently smoked showed a higher ΔE value.

### 3.2. Texture

The ability of the smoking process to preserve fish is due to the synergistic action of salt incorporation, the preservative effect of smoke compounds, and dehydration. This also causes a significant increase in all textural parameters, as demonstrated on other fish species [9,12,26,27,44,47,48]. In our study, as shown in Supplementary Table S3, textural parameters (Young modulus and hardness) increased significantly (*p* < 0.05) after smoking in both treatments. This trend could also be observed in all subsequent smoking processes.

In smoking I, differences in Young’s modulus and hardness were observed in the NaCl samples during storage, and these parameters increased to 0.76 ± 0.11 and 24.83 ± 1.23 on day 14 (*p* < 0.05), respectively. This then decreased and stabilized in the following days of shelf-life (Supplementary Table S3), while in the NaCl/KCl samples, the textural parameters remained constant throughout the shelf-life.

These results highlight that the degradation processes, related to autolytic phenomenon and to the denaturation of protein in the muscle tissue, were more accentuated in the NaCl samples than the NaCl/KCl samples [49].

No significant differences (*p* > 0.05) were observed between the different smoking processes in the early shelf-life times. It was from T21 that differences were observed, in particular, smoking III and V showed significantly higher (*p* < 0.05) hardness values than the other smoking processes.

### 3.3. Water Holding Capacity (WHC)

The water holding capacity measures the ability of the muscle to retain water and it increased significantly (*p* < 0.05) in the smoked fillets compared to the untreated samples without differences between the two treatments (Supplementary Table S4), which is in agreement with results obtained in other studies [9,27].

Significant differences (*p* < 0.05) were found between the fresh fillets and the thawed fillets, with the second ones showing lower values than the first ones, perhaps as a result of the damage due to freezing/thawing [13]. The WHC increased after smoking in all smoking processes and remained stable throughout the storage time without differences between the two treatments in accordance with Fuentes et al. [27], as the result of the increment in salt content positively correlated with the WHC. 

### 3.4. Muscular pH

The pH value of the fresh fillets was significantly different (*p* < 0.05) from those of the thawed fillets as shown in Supplementary Table S5. Soto-Valdez et al. [50] found an increase in the pH during ice storage, probably due to the endogenous proteolytic activity or microbial action that determines an accumulation of alkaline compounds. 

Regarding the smoked samples, the pH was reduced significantly by the smoking process (*p* < 0.05). Indeed, a significant difference between the untreated samples and the smoked samples was observed and this difference remained over the storage time (*p* < 0.05). Regarding smoking I, the samples treated with NaCl tended to show lower pH than those of NaCl/KCl samples at the end of the storage time (*p* < 0.05), as shown by Fuentes et al. [27]. 

These findings, in agreement with those shown in previous studies on smoked fish like dolphinfish, salmon, and sea bass [7,9,27], were probably due to the salting and smoking treatments that determine an increase in the ionic strength. The difference found in the present study between the NaCl and NaCl/KCl samples could have been due to the salt composition, which affected the ionic strength of the intracellular solution. Moreover, pH may be affected by the lack of production of volatile basic components by spoilage bacteria observed in this study that help maintain a constant low value of pH during storage after processing [27,29].

### 3.5. NA, K, and Salt Content 

**Figure 2:**
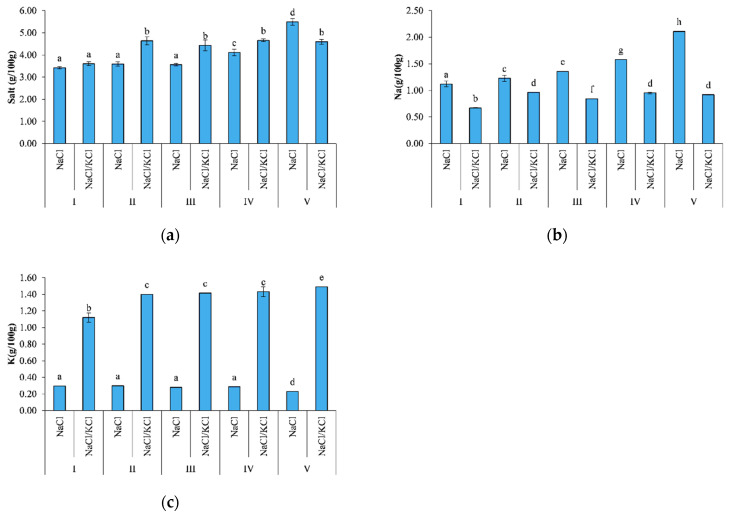
Salt, Na, and K determined in the smoked fillets of *D. labrax*, in the five smoking processes (I, II, III, IV, V). Different letters (a, b, c…) indicate significant differences (*p* < 0.05). (**a**) Salt content; (**b**) Na content; (**c**) K content.

Significant differences (*p* < 0.05) in the sodium content were found between smoking I and smoking V at 1 g/100 g and 2 g/100 g, respectively, in the NaCl samples, and significant differences (*p* < 0.05) were found between the two treatments in all smoking processes, as shown in Figure 2.

The potassium content increased significantly (*p* < 0.05) from 1 g/100 g in smoking I to around 1.5 g/100 g in smoking V with regard to the NaCl/KCl samples, and significant differences (*p* < 0.05) were found between the two treatments in all smoking processes. 

The fillets in smoking V were significantly saltier (*p* < 0.05) than the fresh/smoked fillets, and significant differences (*p* < 0.05) were found between the two different treatments. 

The fish processing led to a reduction in the moisture content and to an increase in the mineral content as observed in previous studies [9,26,27]. In the present study, smoking V showed a higher salt content (*p* < 0.05) compared to the previous ones as far as the NaCl samples were concerned, probably because of the pH and textural changes that inferred the capacity of the muscle to retain water. 

These findings confirmed that salt replacement with KCl may contribute to healthier products, considering that they may also be a source of potassium compared to the traditional product, providing around 30% of the recommended allowances for adults (4.7 g) when both the fresh fillets and the frozen fillets were processed [27,28].

### 3.6. Biochemical Parameters Related to the Shelf-Life

The proximate composition and water activity of fresh and smoked fillets are shown in Table 2. The lipid content showed that *D. labrax* is a medium-fat fish species [51]. The nutritional values remained unchanged following smoking, except for the contents of ash and water, which varied due to water loss. These features were in agreement with those obtained in previous studies for the same fish species [26]. Moreover, as fish processing led to a decrease in aw [9,26,27], even in the present study, aw was reduced after smoking compared to unprocessed products (Table 2) and did not show any difference between all smoking processes carried out with regard to both NaCl and NaCl/KCl samples throughout the storage time (data not shown).

Fatty acid (FA) composition of European sea bass fillets showed no significant difference among salting treatments up to the end of the storage time (T35) (Table 3). This finding confirms that the cold smoking process and the type of salt used in the brining phase of the process do not alter the biochemical and nutritional properties of the sea bass fillets. The mean values (± SD) of the data recorded on the day after smoking (T1) and on day 35 of the storage period related to both salting treatments are shown in Table 3. All lots were characterized by high levels of monounsaturated FAs followed by saturated FAs, omega-6 (*N*-6) polyunsaturated fatty acids (PUFAs), and *N*-3 polyunsaturated fatty acids (PUFAs) (Table 3).

The smoking process was effective on preventing lipid oxidation and the loss of high beneficial compounds such as n-3 PUFAs considering that no difference (*p* > 0.05) was observed between the first day after processing (T1) and the last day of storage. Moreover, no difference was observed between the two salting treatments applied (Table 3).

**Figure 3:**
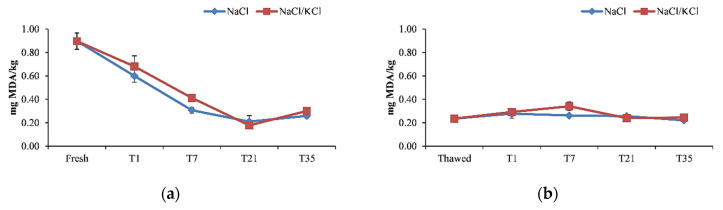

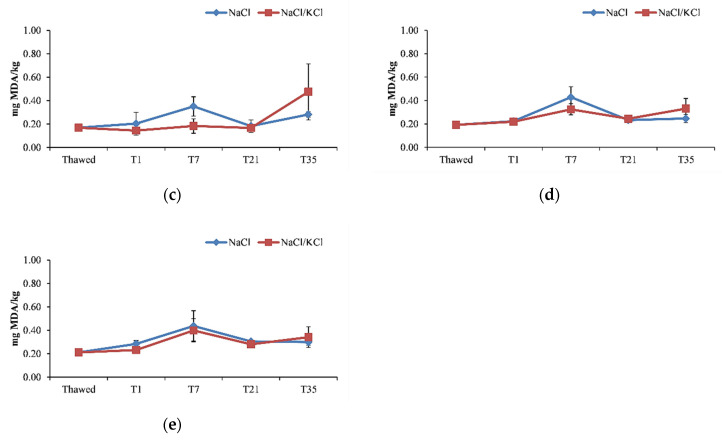
Malondialdehyde (MDA) levels (mg/kg) in the smoked fillets of *D. labrax* during the 35 days of preservation, in the five smoking processes. (**a**) smoking I; (**b**) smoking II; (**c**) smoking III; (**d**) smoking IV; (**e**) smoking V.

Regarding the effectiveness of the smoking process on preserving fish fillets on the loss of high beneficial compounds, the malonaldehyde (MDA) content reduced significantly (*p* < 0.05) after smoking with similar patterns between the NaCl and NaCl/KCl samples (Figure 3).

Indeed, it reduced significantly at T1 from 0.9 mg MDA/kg measured in the raw material, which approached the value of 1 mg MDA/kg, considered as an indicator of the onset of oxidative rancidity in the fresh fish [52]. This value reduced progressively during the storage time (Figure 3). In the other smoking processes, the MDA content was significantly lower in the thawed fillets compared to the fresh fillets and the smoking process contributed to maintain that low value. In general, in the frozen/smoked samples, the MDA content tended to increase slightly at T7, being constant enough during the storage time, and at T35, it was not significantly different between the smoking processes, maintaining well below the 4.5 mg MDA/kg value considered acceptable for smoked fish in the literature [29]. As observed in previous studies [17,21,52]—even in this study—the salting/smoking treatment did not exert any effect on lipid oxidation, as revealed by the MDA content, and could not be used as an indicator of fish spoilage. This should be attributed to the antioxidant activity of the phenolic fraction of the smoke, despite the potential prooxidant effect of salting [21]. The right salting concentration and salting time can also be decisive in avoiding lipid oxidation [29]. Moreover, in the present study, no difference was observed (*p* > 0.05) between NaCl samples and NaCl/KCl samples in terms of MDA content during the 35 days of storage, thus confirming the effectiveness of the partial sodium replacement on preventing lipid oxidation, as shown in other studies [29]. Moreover, vacuum packaging might be effective in preserving frozen fillets and processed products from lipid oxidation, providing more evidence on the effective use of this method of conservation [17].

**Figure 4:**
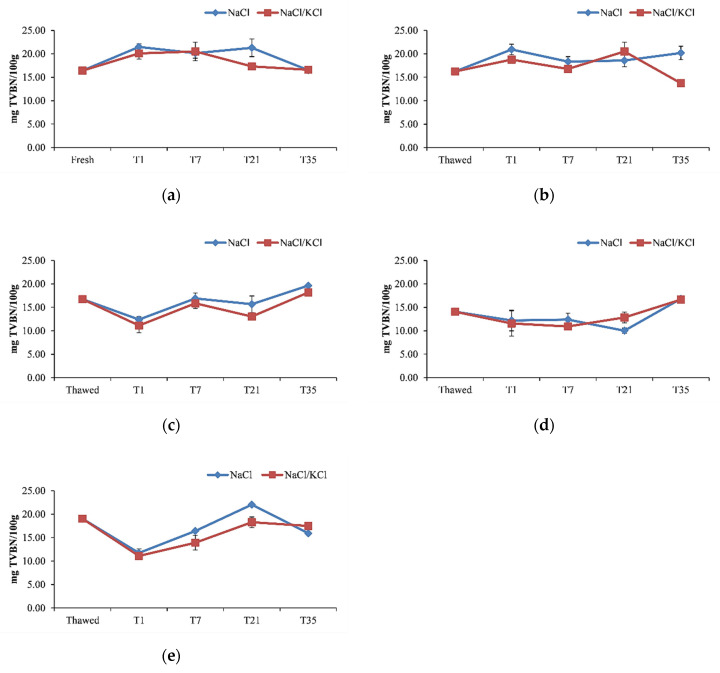
Total volatile basic nitrogen (TVBN) (mg/100 g) in the smoked fillets of *D. labrax* during the 35 days of preservation, in the five smoking processes. (**a**) smoking I; (**b**) smoking II; (**c**) smoking III; (**d**) smoking IV; (**e**) smoking V.

The TVB-N content in the fresh fillets was 16.41 mg/100 g. No difference was observed in the TVB-N content between the fresh fillets and the thawed fillets kept frozen for 30 days (Figure 4).

The TVB-N content increased at T1 as far as the first two smoking processes were concerned, but it reduced significantly after smoking at T1 in the fillets kept frozen for 30, 60, and 90 days, without significant differences between these last three frozen/smoked samples, and after that time, it increased again. As reported in previous studies where the aptitude of frozen fish fillets for cold smoking was investigated [17], a reduction in the level of TVB-N in frozen fillets after salting/smoking was observed most likely because part of the volatile bases produced during frozen storage were dissolved in the brine or volatilized during smoking. The acceptability limit of 30–35 mg *N*/100 g [40] was not exceeded in any of the smoking processes, at any time of the storage period, agreeing with the results obtained previously on vacuum packaged smoked sea bass, which showed values of TVB-N below the above-mentioned limit of up to 42 days of storage [21]. Moreover, the TVB-N may be affected by the reduction in both the spoilage bacteria and the activity of endogenous enzymes due to the salting/smoking processes, which is in agreement with another study dealing with the effect of different smoking flavorings on the quality of farmed European sea bass [53]. The results of this study provide evidence that for up to 30 days of storage, the smoking carried out in the three smoked samples greatly inhibited the formation of spoilage oxygenated metabolites and therefore the growth of the microorganisms responsible for it. Nevertheless, sulfur derivatives were detected in samples smoked with the smoke flavoring with the higher phenol content after 25 days of vacuum packaging at refrigeration temperature.

It has to be highlighted that the presence and proliferation of TMA-N producing bacteria, for instance, Enterobacteriaceae, were prevented by strictly observing the hygiene conditions before and during treatments; moreover, the temperature was monitored during refrigerated storage at 4 °C. Lactobacilli have also been identified as spoilage bacteria that produce sulfuric acid that can cause unpleasant odors, which was not found in this work. In Rizo et al. [54], it was shown that TVB-N reached values that considered the smoked cod product not acceptable after 21 days of storage although bacterial counts led to the consideration that the product was acceptable after 40 days of storage. Another study by Messina et al. [9] therefore took into consideration both the biochemical and microbiological parameters in assessing the effectiveness of cold smoking in extending the shelf-life of smoked dolphinfish fillets to evaluate how cold smoking, carried out under the same conditions observed in the present work, significantly (*p* < 0.05) prevented the proliferation of mesophilic and psychrophilic bacteria, which remained within the acceptable limits (< 10^7^ CFU/g) until the end of its shelf-life, although in this case, the concentration of TVB-N also remained within the acceptable limits up to 21 days of refrigerated storage. Therefore, this evidence supports the fact that despite the microbiological risk due to the low temperatures used in the process, cold smoking, if carried out with appropriate measures (the same ones observed in the present study) has an effective bacteriostatic action that combined with the appropriate storage conditions such as temperature and vacuum packaging does not cause microbial proliferation as indicated by the TVB-N values used among the quality indicators of the smoked product as described above. Moreover, another study by Gómez-Estaca et al. [17] aimed at evaluating the effect of freezing sardine and dolphinfish fillets on the quality of the cold-smoked product did not consider the microbiological parameters among those used.

In the present study, the partial replacement of NaCl with KCl maintained, in general, the TVB-N content during the storage period, which in all of the smoking processes was significantly lower compared to 100% NaCl samples, highlighting the effectiveness of using KCl along with NaCl on maintaining low values of this parameter during the shelf-life.

### 3.7. Sensory Analysis

In order to gain good results by a processing technique that is useful to add value to a seafood product such as cold smoking, it is necessary that the raw material be of good quality. If this assumption is not observed, the quality of the final product can be impaired regardless of the effectiveness of the technique used [55].

The sensory evaluation of raw and treated fillets provides important evidence about the quality of the raw material and the degree of consumer acceptability by analyzing attributes of the product that are directly evaluated by consumers that in the case they were altered, they could compromise their choice and satisfaction. These features, for example, are taste, texture, odor, and color, which could be affected by the smoking process, so the physicochemical parameters of the finished products that would need to be regulated.

The quality of raw fresh fillets was also of good quality, except in smoking V, where the sensory parameters of the thawed fillets were on average evaluated to be of bad quality (Table 4). The sensory evaluation of the raw material showed that the quality of fillets was impaired by freezing in particular, as reported in the literature for salmon [44], and the main parameter altered by freezing was texture, as shown in the present study.

The findings of this study related to the sensory assessment of cold smoked fresh fillets (Supplementary Table S6) showed that the partial replacement of NaCl by KCl did not significantly alter the odor, color, appearance, and taste of the smoked fillets. These results were in accordance with those obtained in previous studies where 50% of sodium replacement by potassium was shown to be feasible for smoked sea bass on the basis of the sensory evaluation [26]. There were significant differences between the smoking processes at T1 (*p* < 0.05). In smoking V, the sensory attributes were considered neutral, unlike the previous smoking processes where they were evaluated with higher scores (4–5) that corresponded to less typical and very typical.

Moreover, it has to be highlighted that the odor maintained high scores that ranged from neutral to typical during refrigerated storage under vacuum packaging, providing that no sulfur compounds were produced, so shows that the treatment was effective on preventing the growth of microorganisms responsible for their formation, like, for instance, *Shewanella putrefaciens* [53].

There were significant differences between the sensory attributes scores of the smoked samples at several times of the storage period considered. It was observed that during the storage time, the fillets of the fifth batch, which were treated in smoking V, showed lower scores compared to the other ones. However, the attributes of the fillets treated in the previous smoking processes maintained higher scores up until 21 days.

In general, no significant differences were found for any of the evaluated attributes depending on the type of salt.

### 3.8. Correlation among Quality Parameters of Raw and Smoked Fillets by Principal Component Analysis 

**Figure 5:**
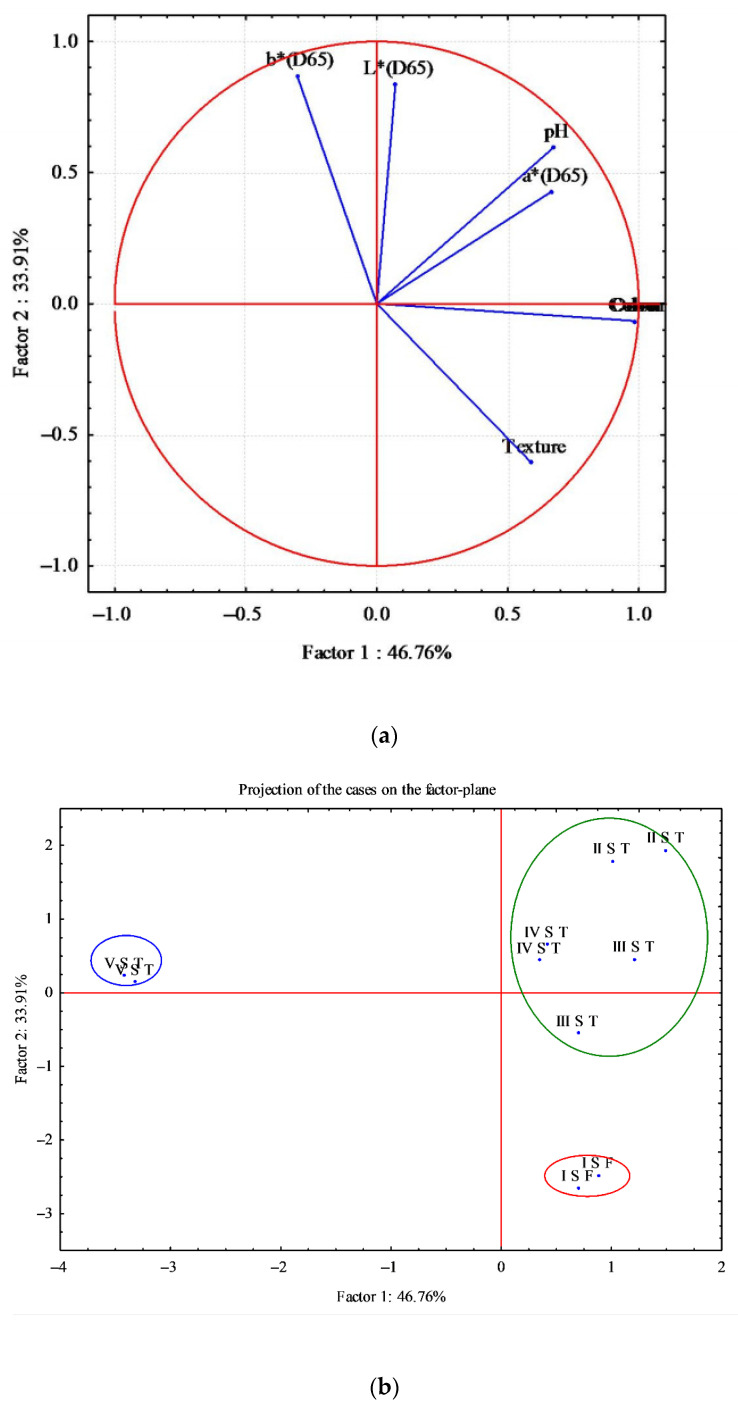
Principal component analysis (PCA) obtained from correlation of seven components (sensory analysis, pH, and color (L*, a*, b*) and 10 cases determined on fresh and thawed fillets of *D. labrax*. (**a**) Correlation circle; (**b**) Factor-plan.

**Figure 6:**
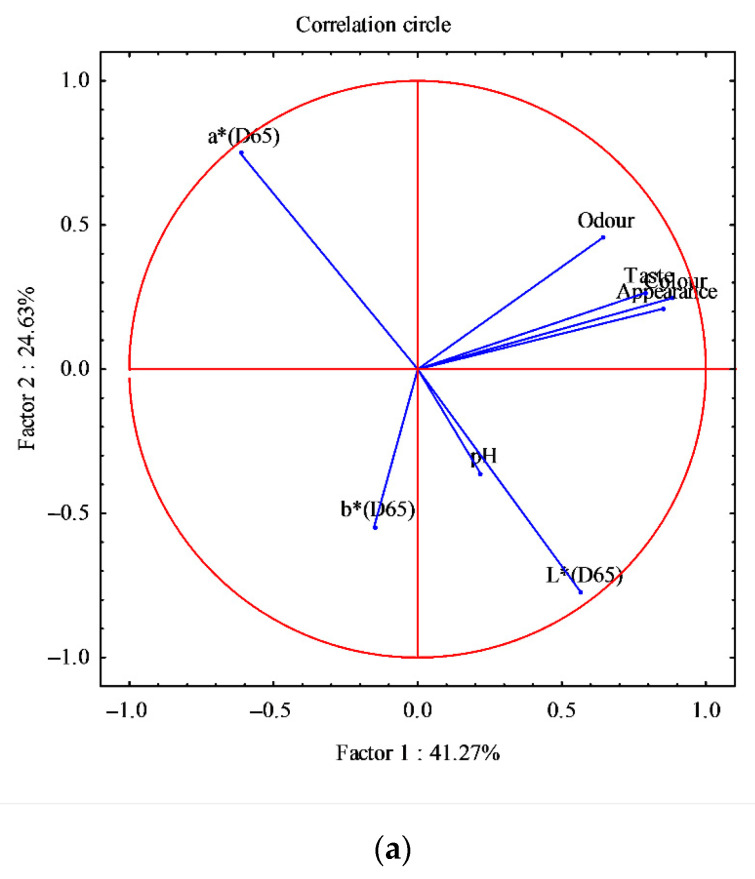

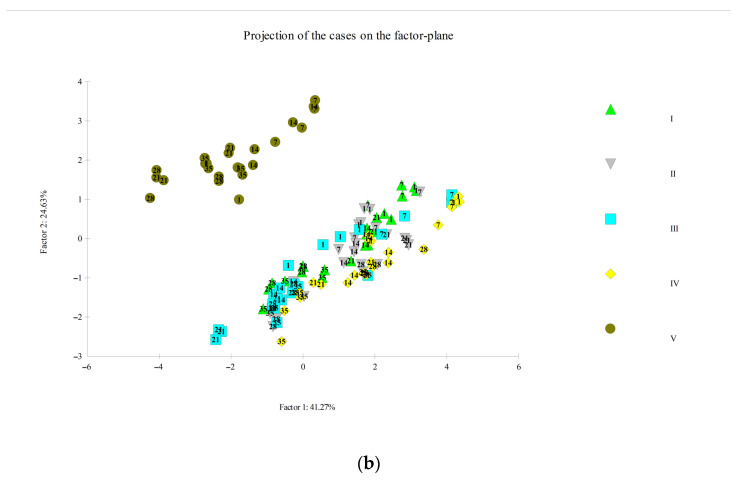
Principal component analysis obtained from the correlation of eight components (sensorial analysis, pH, and color) and 120 cases, determined in the smoked fillets *D. labrax*. (**a**) Correlation circle; (**b**) Factor-plan. In Figure 6b, different symbols, from I to V, indicate the smoking cycles.

The differences observed in sensory and instrumental analysis (color and pH) between fresh and thawed fillets and between smoked fillets were further analyzed through analysis of the main components (PCA) (Figure 5 and Figure 6).

Figure 5 shows the graph obtained from the PCA. Seven variables (sensory analysis: odor, color and texture; color: L*, a* and b*; pH) and 10 cases were analyzed. The analysis generated a small number of linear combinations on the seven variables and only two main components were identified with an eigenvalue greater than 1. These two main components explain 80.67% of the variance of the original variables.

The first main component (Factor 1) explains 46.76% of the variance and the second component 33.91%. The circular correlation between the first two main components is shown in Figure 5a. Figure 5b shows the factorial map of the samples, identifying three very distinct groups; in particular, it was observed that the samples of the last thawing were arranged in another quadrant of the map and were different from the other fresh and thawed samples (Figure 5b).

The fresh samples (I S F) were arranged in the lower quadrant, where the variable that mainly affected their characteristics was the texture (Figure 5a,b). In fact, due to thawing, the thawed samples had a lower score (Table 4), losing their tonicity; moreover, fresh fillets showed greater compactness, recording higher Young modulus values (Table 4).

The PCA was also performed on smoked fillets for all five smoked products; eight variables were identified (sensory analysis: odor, color, appearance and taste; color: L*, a* and b*; pH) and 120 cases. The analysis generated a small number of linear combinations on the eight variables and only three main components with an eigenvalue greater than 1 were identified. These three components explain 79.28% of the variance of the original variables.

The first main component (Factor 1) explains 41.27% of the variance, the second component 24.63%, and the third component 13.38%. The circular correlation has been realized considering the first two main components (Figure 6a); this graph shows how the eight variables were arranged in the experimental case examined. Figure 6b shows the factorial map of the samples, identifying two very distinct groups, where smoking V is clearly separated from the other smoking processes (Figure 6b).

This reflects the result of the previous PCA (Figure 5a,b) where the thawed fillets, used in smoking V, were of inferior quality compared to other fillets, thus affecting the smoking and the final product [55].

## 4. Conclusions

Freezing affected the texture of the sea bass fillets, in particular, the fresh fillets were of better quality in terms of compactness (i.e., Young modulus) compared to the thawed fillets, and the worst ones were fillets thawed after 90 days of frozen storage. The quality of the raw fillets consequently affected the quality of the cold smoked fillets, based on the sensory attributes scores and color evaluation, indicating that sea bass fillets could not be frozen for more than 60 days if they were meant to be treated by cold smoking.

Cold smoking was effective on preventing lipid peroxidation and in maintaining TVB-N values below the threshold for spoilage. Moreover, the type of salt used in the process did not alter the quality of the smoked fillets, providing evidence about using potassium as a sodium replacement when cold smoked sea bass fillets are produced, in order to develop a low-salt fish product.

The findings of the present study provided important evidence on developing a new value-added fish product from aquaculture that can contribute to diversifying the seafood products available to consumers given that the supply of European sea bass fillets often exceeds the demand. In this case, a ready-to-eat product could be developed with important practical applications for the sector considering that the pace of modern life makes this kind of product greater in demand.

## Figures and Tables

**Table 1:** Evaluation of the raw fillets with skin [42].

Object of Assessment	Criteria			
	Freshness Category			
	Extra Score 3	A Score 2	B Score 1	Not Admitted Score 0
External evaluation				
Texture of fillet with skin	Very firm, rigid, elastic	Fairly rigid, firm, slightly less elastic	Slightly soft, less elastic	Soft (flaccid)
Skin odor	Fresh seaweed	Neutral	Slightly sour	Sour
Flesh				
Flesh color	Translucent, smooth, bluish Color around the spinal column: absent	Slightly modified and waxy Color around the spinal column: slightly pink	Slightly opaque and waxy Color around the spinal column: pink	Opaque, yellow Color around the spinal column: red

**Table 2:** Proximate composition in the fresh and smoked fillets of *D. labrax.*

		Smoked Fillets	
	Fresh Fillets	NaCl	NaCl/KCl
Lipids	6.16 ± 2.34 ^AB^	5.93 ± 0.31 ^A^	6.58 ± 0.86 ^B^
Moisture	74.13 ± 1.78 ^A^	72.79 ± 0.3 ^B^	71.3 ± 0.55 ^B^
Ash	1.42 ± 0.21 ^A^	4.14 ± 0.19 ^B^	4.86 ± 0.19 ^B^
Protein	18.29 ± 4.33 ^A^	17.14 ± 1.52 ^B^	17.26 ± 1.6 ^B^
aw	0.987 ± 0.001 ^A^	0.942 ± 0.004 ^B^	0.95 ± 0.004 ^B^

Data within a row, showing different superscript letters ^(A,B)^ are statistically different (*p* < 0.05).

**Table 3:** Fatty acid composition in the fresh and smoked fillets of *D. labrax* at the beginning and at the end of storage time.

		Smoked Fillets			
		T1		T35	
	Fresh Fillets	NaCl	NaCl/KCl	NaCl	NaCl/KCl
SFA	21.29 ± 0.32 ^A^	21.14 ± 0.48 ^A^	20.82 ± 1.59 ^A^	19.96 ± 1.94 ^A^	19.73 ± 1.39 ^A^
MUFA	45.79 ± 2.67 ^A^	45.33 ± 0.43 ^A^	46.11 ± 0.35 ^A^	46.68 ± 2.09 ^A^	46.40 ± 0.96 ^A^
PUFA	32.91 ± 2.99 ^A^	33.54 ± 0.91 ^A^	33.07 ± 1.24 ^A^	33.36 ± 0.15 ^A^	33.87 ± 2.35 ^A^
Tot n-3	15.26 ± 2.74 ^A^	15.75 ± 0.52 ^A^	15.88 ± 0.54 ^A^	15.70 ± 0.86 ^A^	16.05 ± 2.87 ^A^
Tot n-6	16.96 ± 0.23 ^A^	17.04 ± 0.45 ^A^	16.47 ± 0.65 ^A^	16.91 ± 0.65 ^A^	17.08 ± 0.56 ^A^

Data within a row showing the same superscript letter ^(A)^ were not statistically different (*p* < 0.05).

**Table 4:** Sensory analysis measured in fresh and thawed *Dicentrachus labrax* fillets.

Raw Fillet (Smoking)	Texture	Odor	Color	Average
Fresh (I)	3.00 ± 0.00 ^a^	2.00 ± 0.00	1.67 ± 0.47	2.22 ± 0.69
Thawed (II)	2.00 ± 0.00 ^b^	2.00 ± 0.00	2.00 ± 0.00	2.00 ± 0.00
Thawed (III)	1.50 ± 0.71 ^c^	2.00 ± 0.00	1.50 ± 0.71	1.67 ± 0.29
Thawed (IV)	1.00 ± 0.00 ^d^	2.00 ± 0.00	2.00 ± 0.00	1.67 ± 0.58
Thawed (V)	1.00 ± 0.00 ^d^	2.00 ± 0.00	1.25 ± 0.35	1.42 ± 0.52

3–2.5 score: Extra class (extra quality fish); <2.5–1.5 score: A class (good quality fish); <1.5–0.5 score: B class (bad quality fish); <0.5 score ¼ unfit for human consumption. Data within a column showing different superscript letters ^(a, b, c…)^ are statistically different (*p* < 0.05).

## Supplementary Materials

The following are available online at https://www.mdpi.com/2076-2615/11/1/185/s1, Table S1: Color parameters L*, a*, b*; Table S2: Color parameters C*, h, ΔE; Table S3: Texture parameters, Young modulus and hardness; Table S4: Water holding capacity (WHC); Table S5: pH; Table S6: Sensory evaluation of the smoked fillets, odor, color, appearance, and taste.
