# Supplementary material for: Combination of Freezing, Low Sodium Brine, and Cold Smoking on the Quality and Shelf-Life of Sea Bass (Dicentrarchus labrax L.) Fillets as a Strategy to Innovate the Market of Aquaculture Products
Source: Animals (Basel). 2021 Jan 14;11(1):185. doi: 10.3390/ani11010185 (PMC7830399; doi:10.3390/ani11010185)
Supplement: Supplementary file 1 [file animals-11-00185-s001.pdf]

animals

MDPI

Supplementary materials: Combination of freezing, cold smoking and low sodium brine on the quality and shelf-life of sea bass (*Dicentrarchus labrax* L.) filletts, as a strategy to innovate the market of aquaculture products.

Concetta Maria Messina, Giovanna Ficano, Rosaria Arena, Laura La barbera, Maria Morghese, Andrea Santulli

Table 1. Parameters related to color—L* a*, and b*— measured in the smoked fillets of *D. labrax* during the 35 days of preservation in the five smoking processes (I, II, III, IV,V).

		I	II	III	IV	V
**Day of storage**	**Type of brine solution**			**L*(D65)**		
Fresh/thawed		41,86±0,86$^{ab/A}$	51,39±2,48$^{a/B}$	44,49±3,12$^{a/AC}$	46,19±1,31$^{a/C}$	46,67±2,57$^{ab/C}$
1	NaCl	39,95±1,70$^{a/A}$	45,53±2,85$^{b/B}$	44,38±2,96$^{a/B}$	40,70±2,15$^{c/A}$	48,31±1,01$^{ab/B}$
	NaCl/KCl	42,18±0,96$^{ab/A}$	49,82±3,76$^{ab/B}$	43,29±2,89^{aA}	41,26±0,47$^{bc/A}$	45,09±2,54$^{a/A}$
7	NaCl	41,26±0,80$^{ab/A}$	46,60±2,75$^{ab/AB}$	44,60±2,02^{aAB}	41,25±2,37$^{bc/A}$	46,80±2,64$^{ab/B}$
	NaCl/KCl	39,89±1,03$^{a/A}$	45,23±2,25$^{b/C}$	44,59±1,80^{aBC}	41,69±1,31$^{bc/AB}$	46,93±2,28$^{ab/C}$
14	NaCl	42,31±1,78$^{ab/A}$	45,28±1,10$^{b/B}$	43,11±3,17^{aA}	44,28±1,22$^{abc/B}$	46,42±2,99$^{ab/B}$
	NaCl/KCl	43,23±0,95$^{b/A}$	46,29±2,76$^{ab/B}$	45,24±3,01^{bB}	44,03±2,09$^{abc/AB}$	46,10±3,07$^{ab/B}$
21	NaCl	44,14±2,12$^{bc/AB}$	47,09±1,46$^{ab/B}$	42,95±2,01^{aA}	45,74±2,32$^{ab/AB}$	47,32±1,44$^{ab/B}$
	NaCl/KCl	43,59±1,58$^{b/A}$	45,60±2,40$^{b/A}$	45,56±1,42^{bA}	43,71±3,51$^{abc/A}$	46,51±0,92$^{ab/A}$
28	NaCl	43,41±1,61$^{b/A}$	45,37±1,50^{bA}	45,75±1,56^{bA}	44,79±2,47$^{abc/A}$	47,53±3,01$^{ab/A}$
	NaCl/KCl	42,29±0,63$^{ac/A}$	48,55±1,06$^{ab/B}$	46,94±1,91^{abB}	47,39±1,26$^{a/B}$	48,84±3,02$^{ab/B}$
35	NaCl	46,28±1,20$^{c/A}$	45,10±1,46$^{b/A}$	47,21±1,08^{cA}	46,81±1,30$^{a/A}$	51,15±2,41$^{b/B}$
	NaCl/KCl	44,29±1,76$^{bc/A}$	46,89±2,58$^{ab/A}$	46,16±1,52^{abA}	46,49±2,71$^{a/A}$	48,37±1,94$^{ab/A}$
				a*(D65)		
Fresh/thawed		-1,77±0,40^{aA}	-1,01±0,31$^{a/BC}$	-0,39±1,38$^{a/C}$	-1,30±0,61$^{ab/B}$	-1,62±1,28$^{a/AB}$
1	NaCl	-0,95±0,56^{bA}	-2,20±0,28$^{b/B}$	-1,84±0,18$^{ab/B}$	-1,41±0,49$^{ab/AB}$	-3,13±0,66$^{ab/C}$
	NaCl/KCl	-1,02±0,63^{bA}	-3,73±0,48$^{bc/B}$	-2,09±0,59$^{ab/AB}$	-0,95±1,43$^{a/A}$	-2,43±1,27^{abAB}
7	NaCl	-1,28±0,97^{abA}	-3,19±1,06$^{bc/CD}$	-2,70±0,81$^{b/BC}$	-2,05±0,69$^{abc/AB}$	-4,12±0,53$^{b/D}$
	NaCl/KCl	-0,90±1,01^{bA}	-3,55±0,65$^{bc/BC}$	-3,06±0,40$^{b/BC}$	-1,99±0,89$^{abc/AB}$	-3,97±0,45$^{ab/C}$
14	NaCl	-1,72±1,49^{aA}	-3,55±0,88$^{bc/B}$	-2,79±0,87$^{b/B}$	-1,79±1,33$^{abc/A}$	-3,34±0,46$^{ab/B}$
	NaCl/KCl	-2,03±1,05^{cA}	-3,56±1,01$^{bc/B}$	-2,84±0,67$^{b/B}$	-1,86±0,77$^{abc/A}$	-3,64±1,23$^{ab/B}$
21	NaCl	-1,80±1,45^{acA}	-3,96±1,01$^{bc/B}$	-1,79±0,80$^{ab/A}$	-2,23±1,10$^{abc/A}$	-3,89±0,72$^{ab/B}$
	NaCl/KCl	-1,20±1,59^{abA}	-3,23±1,02$^{bc/B}$	-3,44±0,72$^{b/B}$	-3,17±0,44$^{bc/B}$	-3,91±0,77$^{ab/B}$
28	NaCl	-1,38±1,53^{abA}	-3,95±0,20$^{bc/B}$	-2,38±1,66$^{b/B}$	-2,06±0,71$^{abc/B}$	-2,81±1,81$^{ab/B}$
	NaCl/KCl	-1,67±1,43^{aA}	-4,17±0,67$^{c/B}$	-3,36±0,60$^{b/B}$	-2,95±1,02$^{abc/AB}$	-4,35±0,36$^{ab/B}$
35	NaCl	-2,98±0,63^{dA}	-3,35±0,58$^{bc/A}$	-3,59±0,77$^{b/A}$	-3,30±0,81$^{bc/A}$	-2,91±1,37$^{ab/A}$
	NaCl/KCl	-1,46±0,95^{abA}	-3,32±0,71$^{bc/B}$	-2,81±1,75$^{b/B}$	-3,81±0,61$^{c/B}$	-3,13±1,06$^{ab/B}$
				b*(D65)		
Fresh/thawed		-4,78±0,55$^{ac/A}$	-2,02±1,40$^{ac/B}$	-1,27±0,92$^{ac/B}$	-1,71±0,86$^{ad/B}$	-0,48±0,90$^{ab/B}$
1	NaCl	-5,79±0,68$^{a/A}$	-4,86±0,79$^{b/AB}$	-3,47±1,12$^{a/BC}$	-5,05±1,00$^{b/AB}$	-2,46±0,79$^{a/C}$
	NaCl/KCl	-7,75±1,72$^{b/A}$	-6,53±1,20$^{b/AB}$	-5,91±0,69$^{b/AB}$	-4,30±1,03$^{bc/BC}$	-2,49±1,24$^{a/C}$

7	NaCl	-5,89±0,51$^{ac//A}$	-2,03±1,24$^{ac/B}$	-2,14±0,99$^{ac/B}$	-3,48±1,05$^{abc/B}$	2,07±1,33$^{cd/C}$
	NaCl/KCl	-5,18±1,95$^{ac/A}$	-2,61±0,62$^{a/B}$	-3,21±1,00$^{a/AB}$	-3,46±0,85$^{abc/AB}$	1,17±1,87$^{bc/C}$
14	NaCl	-4,63±0,68$^{ac/A}$	-0,74±0,57$^{acd/B}$	-2,49±0,85$^{ac/C}$	-1,98±1,02$^{ad/C}$	2,47±0,36$^{cde/D}$
	NaCl/KCl	-4,16±0,57$^{acd/A}$	-0,34±1,66$^{acd/BD}$	-1,67±1,53$^{ac/BC}$	-2,59±0,84$^{acd/C}$	1,13±0,70$^{bc/D}$
21	NaCl	-3,31±0,81$^{acd/A}$	0,87±1,07$^{d/C}$	-1,15±1,28$^{ac/B}$	-1,51±0,59$^{ad/B}$	4,15±0,44$^{def/D}$
	NaCl/KCl	-4,63±1,00$^{ac/A}$	0,16±1,78$^{cd/B}$	-2,51±1,21$^{ac/C}$	-1,54±0,98$^{ad/BC}$	4,27±1,57$^{def/D}$
28	NaCl	-1,90±0,62$^{d/A}$	1,09±1,10$^{d/B}$	-1,62±0,40$^{ac/A}$	-0,73±0,90$^{d/A}$	6,83±1,52$^{g/C}$
	NaCl/KCl	-3,52±1,32$^{acd/A}$	1,18±0,72$^{d/B}$	-0,55±0,72$^{cd/BC}$	-1,47±1,75$^{ad/C}$	3,43±0,93$^{cde/D}$
35	NaCl	-2,78±0,93$^{cd/A}$	0,67±1,46$^{d/B}$	-1,35±0,72$^{ac/AB}$	-1,12±1,06$^{ad/AB}$	6,48±2,17$^{fg/C}$
	NaCl/KCl	-3,53±1,54$^{acd/A}$	0,51±1,22$^{d/B}$	0,88±1,77$^{d/B}$	-0,64±1,66$^{d/B}$	4,89±1,29$^{efg/C}$

Data within a column, showing different superscript letters (a, b, c…), are statistically different (P<0.05). Data within a row, showing different superscript letters (A, B, C…), are statistically different (P<0.05)

Table 2. Parameters related to color—C*, h, and ΔE — measured in the smoked fillets of *D. labrax* during the 35 days of preservation in the five smoking processes (I, II, III, IV, V).

		I	II	III	IV	V
**Day of storage**	**Type of brine solution**			**C*(D65)**		
Fresh/thawed		5,10±0,60$^{ab/A}$	2,42±1,04$^{a/B}$	1,65±1,20$^{a/B}$	2,16±1,01$^{a/B}$	1,89±1,23$^{a/B}$
1	NaCl	5,88±0,72$^{bc/A}$	5,34±0,82$^{b/A}$	3,94±1,07$^{ac/B}$	5,28±0,91$^{b/A}$	4,00±0,91$^{bc/B}$
	NaCl/KCl	7,83±1,76$^{c/A}$	7,54±1,05$^{c/A}$	6,28±0,83$^{b/AB}$	4,55±1,13$^{ab/BC}$	3,54±1,61$^{b/C}$
7	NaCl	6,09±0,47$^{bc/A}$	3,84±1,43$^{ab/AB}$	3,46±1,21$^{ac/B}$	4,06±1,17$^{ab/B}$	4,74±0,66$^{bc/AB}$
	NaCl/KCl	5,37±1,81$^{ab/A}$	4,42±0,78$^{ab/B}$	4,49±0,76$^{c/B}$	4,01±1,15$^{ab/B}$	4,45±0,25$^{bc/B}$
14	NaCl	5,05±1,07$^{ab/A}$	3,65±0,92$^{ab/B}$	3,76±1,16$^{ac/B}$	2,85±1,22$^{a/C}$	4,16±0,54$^{bc/AB}$
	NaCl/KCl	4,70±0,69$^{ab/A}$	3,84±1,13$^{ab/B}$	3,45±1,13$^{ac/B}$	3,27±0,80$^{ab/B}$	3,91±1,01$^{bc/B}$
21	NaCl	3,95±0,95$^{ab/A}$	4,19±0,82$^{ab/A}$	2,32±1,05$^{ac/A}$	2,77±1,00$^{a/A}$	5,69±0,80$^{bcd/B}$
	NaCl/KCl	4,94±1,22$^{ab/AB}$	3,64±0,69$^{ab/A}$	4,33±1,05$^{ac/AB}$	3,58±0,78$^{ab/A}$	5,96±0,60$^{cd/B}$
28	NaCl	2,54±1,23$^{ab/A}$	4,21±0,21$^{ab/A}$	3,04±1,26$^{ac/A}$	2,36±0,51$^{a/A}$	7,59±1,29$^{d/B}$
	NaCl/KCl	4,03±1,57$^{ab/A}$	4,38±0,58$^{ab/A}$	3,46±0,60$^{ac/A}$	3,53±1,40$^{ab/A}$	5,59±0,57$^{bcd/B}$
35	NaCl	4,11±0,93$^{ab/A}$	3,60±0,86$^{ab/A}$	3,86±0,95$^{ac/A}$	3,59±0,91$^{ab/A}$	7,30±1,65$^{d/B}$
	NaCl/KCl	3,83±1,76$^{a/A}$	3,52±0,72$^{ab/A}$	3,27±1,86$^{ac/A}$	4,11±0,68$^{ab/A}$	5,96±0,57$^{cd/B}$
				**h (D65)**		
Fresh/thawed		249,77±3,68$^{ab/A}$	232,97±30,28$^{a/B}$	270,47±45,03$^{a/C}$	232,30±9,48$^{abc/B}$	211,97±55,35$^{a/D}$
1	NaCl	260,82±4,90$^{b/A}$	245,55±2,22$^{a/BC}$	240,94±5,44$^{abc/C}$	253,75±7,06$^{bc/AB}$	217,74±6,78$^{a/D}$
	NaCl/KCl	262,72±3,56$^{b/A}$	239,83±6,14$^{a/AB}$	250,88±3,78$^{ab/A}$	261,41±19,60$^{c/A}$	226,26±15,49$^{a/B}$
7	NaCl	257,83±9,45$^{ab/A}$	209,68±12,81$^{abc/B}$	216,46±9,01$^{bcd/B}$	239,52±6,85$^{abc/C}$	154,45±15,64$^{bc/D}$
	NaCl/KCl	257,87±13,53$^{ab/A}$	216,23±5,80$^{ab/C}$	225,33±10,61$^{bcd/BC}$	241,17±6,32$^{abc/AB}$	163,52±24,76$^{b/D}$
14	NaCl	251,72±14,40$^{ab/A}$	191,39±7,75$^{bcd/B}$	221,83±6,20$^{bcd/A}$	235,50±31,73$^{abc/A}$	143,53±3,36$^{bc/C}$

	NaCl/KCl	244,56±12,40$^{ab/A}$	180,04±24,60$^{cd/CD}$	206,12±21,54$^{bcd/BC}$	233,83±15,47$^{abc/AB}$	160,58±14,85$^{b/D}$
21	NaCl	242,59±20,50$^{ab/A}$	165,58±17,47$^{d/B}$	208,77±29,84$^{bcd/A}$	217,03±15,62$^{abd/A}$	132,91±2,31$^{bc/C}$
	NaCl/KCl	258,09±17,83$^{ab/A}$	173,54±32,77$^{cd/B}$	214,57±12,$^{70bcd/C}$	204,32±11,67$^{ad/C}$	133,58±16,20$^{bc/D}$
28	NaCl	242,99±30,18$^{ab/A}$	164,85±15,05$^{d/}$	224,44±31,23$^{bc/Ad}$	199,35±25,71$^{ad/AB}$	112,92±15,28$^{c/C}$
	NaCl/KCl	249,82±21,30$^{ab/A}$	163,64±10,19$^{d/BD}$	189,63±12,13$^{d/BC}$	198,08±30,44$^{ad/C}$	142,16±8,34$^{bc/D}$
35	NaCl	222,28±8,77$^{a/A}$	171,96±21,43$^{cd/B}$	199,78±6,51$^{cd/A}$	198,30±15,28$^{ad/A}$	116,51±14,41$^{bc/C}$
	NaCl/KCl	248,78±6,11$^{ab/A}$	173,78±21,10$^{cd/B}$	158,26±31,27$^{e/B}$	186,54±24,14$^{d/B}$	123,59±15,32$^{bc/C}$
				**ΔE**		
Fresh/thawed		0,00±0,00^{a}	0,00±0,00^{a}	0,00±0,00^{a}	0,00±0,00^{a}	0,00±0,00^{a}
1	NaCl	2,70±1,01$^{bc/A}$	6,87±2,09$^{b/B}$	3,65±1,29$^{b/A}$	6,64±1,44$^{b/B}$	3,21±0,43$^{b/A}$
	NaCl/KCl	3,33±1,48$^{bc/A}$	6,38±1,33$^{b/B}$	5,60±1,35$^{b/B}$	5,79±0,32$^{bc/B}$	3,38±2,01$^{b/A}$
7	NaCl	1,75±0,46$^{b/A}$	5,56±2,45$^{b/C}$	3,18±0,62$^{b/AB}$	5,35±2,55$^{bc/C}$	4,36±0,83$^{b/B}$
	NaCl/KCl	2,86±1,15$^{bc/A}$	6,78±2,02$^{b/B}$	3,75±0,50$^{b/A}$	4,93±1,59$^{bcd/A}$	3,86±0,42$^{b/A}$
14	NaCl	2,09±0,59$^{b/A}$	6,78±1,24$^{b/B}$	3,88±1,93$^{b/AB}$	2,60±0,77$^{ef/A}$	4,24±0,98$^{b/B}$
	NaCl/KCl	1,97±0,54$^{bc/A}$	6,37±2,07$^{b/B}$	3,91±0,63$^{b/A}$	2,82±1,64$^{def/AB}$	3,85±0,99$^{b/A}$
21	NaCl	3,37±1,37$^{bc/A}$	6,16±1,00$^{b/B}$	2,59±1,77$^{b/A}$	2,28±1,21$^{f/A}$	5,35±0,74$^{bc/B}$
	NaCl/KCl	2,54±1,37$^{bc/A}$	6,89±2,02$^{b/B}$	3,87±0,19$^{b/A}$	4,05±2,10$^{cdef/A}$	5,47±1,00$^{bc/B}$
28	NaCl	3,83±0,51$^{c/A}$	7,49±1,10$^{b/B}$	3,09±0,43$^{b/A}$	2,64±1,67$^{ef/A}$	8,04±1,65$^{d/B}$
	NaCl/KCl	1,90±1,32$^{bc/A}$	5,41±0,84$^{b/B}$	4,24±1,01$^{b/B}$	2,81±0,91$^{ef/A}$	5,76±1,57$^{bc/B}$
35	NaCl	5,15±0,84$^{d/A}$	7,33±1,64$^{b/B}$	4,34±0,92$^{b/A}$	2,69±0,36$^{ef/C}$	8,62±2,61$^{d/B}$
	NaCl/KCl	3,15±1,80$^{bc/A}$	6,03±1,68$^{b/B}$	4,25±1,45$^{b/B}$	3,82±1,01$^{cde/A}$	6,14±1,29$^{c/B}$

Data within a column, showing different superscript letters (a, b, c…), are statistically different (P<0.05). Data within a row, showing different superscript letters (A, B, C…), are statistically different (P<0.05)

Table 3. Parameters related to texture—Young Modulus (N/mm2) and Hardness (N)— measured in the smoked fillets of *D. labrax* during the 35 days of preservation in the five smoking processes (I, II, III ,IV ,V).

		I	II	III	IV	V
**Day of storage**	**Type of brine solution**			**Young Modulus (N/mm²)**		
Fresh/thawed		0,27±0,01$^{a/A}$	0,28±0,03^{aA}	0,27±0,03$^{a/A}$	0,22±0,09$^{a/AB}$	0,20±0,02$^{a/B}$
1	NaCl	0,55±0,00$^{b/AB}$	0,39±0,09^{bA}	0,39±0,08$^{ab/A}$	0,38±0,05$^{b/A}$	0,60±0,20$^{b/B}$
	NaCl/KCl	0,39±0,03$^{ab/A}$	0,38±0,04^{bA}	0,48±0,02$^{ab/B}$	0,41±0,04$^{b/AB}$	0,41±0,10$^{bc/AB}$
7	NaCl	0,43±0,13$^{ab/A}$	0,44±0,03^{cA}	0,48±0,01$^{ab/A}$	0,46±0,12$^{b/A}$	0,45±0,03$^{bc/A}$
	NaCl/KCl	0,55±0,26$^{b/A}$	0,50±0,31^{cAB}	0,54±0,14$^{b/A}$	0,45±0,03$^{b/B}$	0,49±0,04$^{bc/B}$
14	NaCl	0,76±0,11$^{c/A}$	0,48±0,12^{cB}	0,47±0,00$^{ab/B}$	0,36±0,04$^{ab/B}$	0,42±0,01$^{bc/B}$
	NaCl/KCl	0,39±0,01$^{ab/A}$	0,63±0,18^{dB}	0,46±0,05$^{ab/A}$	0,36±0,03$^{ab/A}$	0,55±0,08$^{bc/B}$
21	NaCl	0,34±0,03$^{ab/A}$	0,37±0,00^{bA}	0,46±0,01$^{ab/B}$	0,33±0,02$^{ab/A}$	0,38±0,02$^{c/A}$
	NaCl/KCl	0,38±0,01$^{ab/AB}$	0,42±0,00^{cB}	0,37±0,07$^{ab/AB}$	0,31±0,03$^{ab/A}$	0,40±0,00$^{bc/AB}$

Table 4. Water holding capacity (WHC) variations measured in the smoked fillets of *D. labrax* during the 35 days of preservation in the five smoking processes (I, II, III, IV, V).

		I	II	III	IV	V
**Day of storage**	**Type of brine solution**			**WHC**		
Fresh/thawed		0,88±0,00$^{a/A}$	0,80±0,03$^{a/B}$	0,74±0,04$^{a/C}$	0,82±0,02$^{a/B}$	0,78±0,03$^{a/B}$
1	NaCl	0,96±0,02$^{b/A}$	0,96±0,01$^{b/A}$	0,96±0,01$^{b/A}$	0,97±0,00$^{bc/A}$	0,96±0,00^{bcA}
	NaCl/KCl	0,98±0,00$^{b/A}$	0,95±0,01$^{b/A}$	0,92±0,01$^{b/B}$	0,95±0,04$^{c/A}$	0,98±0,00$^{c/A}$
7	NaCl	0,97±0,00$^{b/A}$	0,97±0,01$^{b/A}$	0,94±0,04$^{b/B}$	0,98±0,00$^{c/A}$	0,98±0,00$^{c/A}$
	NaCl/KCl	0,96±0,00$^{b/A}$	0,95±0,00$^{b/A}$	0,95±0,01$^{b/A}$	0,98±0,00$^{bc/A}$	0,98±0,00$^{c/A}$
14	NaCl	0,97±0,01$^{b/A}$	0,96±0,00$^{b/A}$	0,94±0,08$^{b/A}$	0,97±0,01$^{bc/A}$	0,98±0,00$^{c/A}$
	NaCl/KCl	0,97±0,01$^{b/A}$	0,96±0,00$^{b/A}$	0,95±0,02$^{b/A}$	0,98±0,01$^{bc/A}$	0,98±0,01$^{c/A}$
21	NaCl	0,94±0,01$^{b/A}$	0,93±0,02$^{b/A}$	0,97±0,01$^{b/B}$	0,95±0,02$^{b/AB}$	0,97±0,00$^{bc/B}$
	NaCl/KCl	0,96±0,01$^{b/A}$	0,89±0,05$^{b/B}$	0,90±0,04$^{b/B}$	0,97±0,01$^{bc/A}$	0,97±0,01$^{bc/A}$
28	NaCl	0,86±0,08$^{a/A}$	0,92±0,06$^{b/B}$	0,93±0,03$^{b/B}$	0,98±0,01$^{bc/C}$	0,96±0,00$^{b/C}$
	NaCl/KCl	0,94±0,04$^{b/A}$	0,91±0,06$^{b/B}$	0,92±0,04$^{b/B}$	0,96±0,02$^{bc/A}$	0,96±0,00$^{b/A}$
35	NaCl	0,95±0,00$^{b/A}$	0,95±0,01$^{b/A}$	0,95±0,02$^{b/A}$	0,96±0,00$^{bc/A}$	0,97±0,02$^{bc/A}$
	NaCl/KCl	0,93±0,02$^{b/A}$	0,92±0,09$^{b/A}$	0,91±0,04$^{b/A}$	0,95±0,01$^{b/B}$	0,96±0,00$^{b/B}$
28	NaCl	0,39±0,00$^{ab/A}$	0,29±0,03^{aB}	0,31±0,08$^{a/AB}$	0,31±0,06$^{ab/AB}$	0,35±0,03$^{c/AB}$
	NaCl/KCl	0,42±0,00$^{ab/A}$	0,37±0,05^{bBC}	0,35±0,02$^{ab/CD}$	0,31±0,01$^{ab/D}$	0,39±0,00$^{c/AB}$
35	NaCl	0,37±0,01$^{ab/A}$	0,33±0,04^{abAB}	0,28±0,01$^{a/B}$	0,33±0,04$^{ab/AB}$	0,44±0,04$^{bc/C}$
	NaCl/KCl	0,41±0,00$^{ab/AB}$	0,33±0,00^{abA}	0,37±0,06$^{ab/AB}$	0,34±0,03$^{ab/A}$	0,47±0,01$^{bc/B}$
				**Hardness (N)**		
Fresh/thawed		7,70±1,68$^{a/A}$	9,78±0,80$^{a/B}$	8,69±1,70$^{a/B}$	7,11±4,53$^{a/AB}$	8,30±1,40$^{a/B}$
1	NaCl	16,99±3,11$^{bc/A}$	19,58±0,18$^{b/B}$	15,79±1,31$^{ab/A}$	18,00±2,27$^{cde/AB}$	20,55±1,46$^{bc/B}$
	NaCl/KCl	10,84±1,96$^{b/A}$	17,11±2,24$^{ab/B}$	18,37±0,23$^{b/B}$	20,11±1,13$^{de/B}$	17,98±2,97$^{bc/B}$
7	NaCl	17,87±3,68$^{c/A}$	18,12±1,26$^{b/A}$	24,01±1,54$^{b/B}$	22,19±3,94$^{e/B}$	18,77±2,73$^{bc/A}$
	NaCl/KCl	17,18±3,41$^{bc/A}$	18,18±6,60$^{b/A}$	22,92±6,10$^{b/B}$	19,02±2,00$^{cde/AB}$	23,10±2,39$^{b/B}$
14	NaCl	24,83±1,23$^{d/A}$	20,80±2,04$^{b/B}$	18,67±2,80$^{b/B}$	19,62±3,79$^{cde/B}$	19,15±0,14$^{bc/B}$
	NaCl/KCl	15,85±2,72$^{bc/A}$	18,15±1,14$^{b/A}$	23,01±3,40$^{b/A}$	18,70±0,36$^{cde/AB}$	26,64±3,70$^{c/B}$
21	NaCl	14,53±1,91$^{bc/A}$	12,67±0,59$^{ab/A}$	19,98±0,78$^{b/B}$	10,39±0,89$^{ab/C}$	17,07±0,49$^{b/D}$
	NaCl/KCl	17,35±1,30$^{bc/AC}$	15,40±0,44$^{ab/A}$	17,51±0,46$^{b/AC}$	12,44±2,55$^{abc/B}$	20,06±1,57$^{bc/C}$
28	NaCl	14,73±0,12$^{bc/A}$	16,02±1,89$^{ab/AB}$	15,57±3,21$^{ab/AB}$	14,98±1,29$^{cde/AB}$	18,19±1,40$^{bc/B}$
	NaCl/KCl	18,99±0,10$^{c/AB}$	18,67±1,33$^{b/AB}$	16,24±0,87$^{ab/A}$	19,75±0,42$^{cd/B}$	18,13±0,24$^{bc/AB}$
35	NaCl	12,87±0,26$^{bc/A}$	15,22±0,20$^{ab/A}$	14,57±1,11$^{ab/A}$	14,18±4,07$^{bcd/A}$	20,53±0,73$^{bc/B}$
	NaCl/KCl	14,50±3,10$^{bc/A}$	14,47±0,82$^{ab/A}$	15,05±0,62$^{ab/A}$	18,31±0,64$^{bcde/B}$	19,26±0,90$^{bc/B}$

Data within a column, showing different superscript letters (a, b, c…), are statistically different ($P < 0.05$). Data within a

Data within a column, showing different superscript letters (a, b, c…), are statistically different ($P<0.05$). Data within a row, showing different superscript letters (A, B, C…), are statistically different ($P<0.05$).

Table 5. pH variations measured in the smoked fillets D. labrax during the 35 days of preservation in the five smoking processes (I, II, III, IV, V).

		I	II	III	IV	V
**Day of storage**	**Type of brine solution**			**pH**		
Fresh/thawed		6,29±0,00$^{a/A}$	6,53±0,11$^{a/B}$	6,45±0,06$^{a/B}$	6,44±0,04$^{ab/B}$	6,24±0,00$^{a/A}$
1	NaCl	5,94±0,13$^{bc/A}$	5,49±0,07$^{b/B}$	5,64±0,01$^{a/A}$	6,04±0,02$^{ab/A}$	5,69±0,06$^{a/C}$
	NaCl/KCl	6,02±0,03$^{bc/A}$	5,50±0,05$^{b/B}$	6,04±0,02$^{a/A}$	6,05±0,04$^{ab/A}$	5,93±0,10$^{a/C}$
7	NaCl	5,70±0,02$^{e/A}$	5,39±0,11$^{b/B}$	5,78±0,05$^{d/C}$	5,96±0,05$^{a/D}$	5,62±0,01$^{c/E}$
	NaCl/KCl	6,07±0,14$^{b/A}$	5,39±0,07$^{b/B}$	5,76±0,05$^{d/C}$	6,00±0,01$^{ab/A}$	5,77±0,01$^{b/C}$
14	NaCl	5,89±0,11$^{d/A}$	5,45±0,02$^{b/B}$	5,90±0,00$^{bc/A}$	6,02±0,01$^{ab/C}$	5,61±0,04$^{c/D}$
	NaCl/KCl	5,83±0,04$^{d/A}$	5,56±0,01$^{b/B}$	5,91±0,12$^{ab/A}$	6,03±0,02$^{abc/C}$	5,83±0,05$^{b/A}$
21	NaCl	5,68±0,10$^{e/A}$	5,66±0,01$^{b/A}$	5,93±0,06$^{ab/B}$	5,97±0,13$^{a/B}$	5,65±0,03$^{c/A}$
	NaCl/KCl	5,92±0,04$^{cd/A}$	5,66±0,02$^{b/B}$	5,93±0,01$^{ab/A}$	6,11±0,03$^{c/C}$	5,65±0,19$^{c/B}$
28	NaCl	5,64±0,06$^{e/A}$	5,77±0,01$^{b/B}$	5,89±0,07$^{bc/C}$	5,96±0,09$^{a/D}$	5,43±0,07$^{d/E}$
	NaCl/KCl	5,82±0,02$^{d/A}$	5,76±0,01$^{b/B}$	5,81±0,02$^{cd/A}$	6,04±0,05$^{abc/C}$	5,82±0,02$^{b/A}$
35	NaCl	5,89±0,00$^{d/A}$	5,71±0,12$^{b/B}$	5,89±0,05$^{bc/A}$	5,99±0,01$^{ab/A}$	5,42±0,18$^{d/C}$
	NaCl/KCl	6,00±0,03$^{bc/A}$	5,72±0,02$^{b/B}$	5,86±0,05$^{bc/C}$	6,07±0,05$^{bc/D}$	5,71±0,02$^{b/B}$

Data within a column, showing different superscript letters (a, b, c…), are statistically different ($P<0.05$). Data within a row, showing different superscript letters (A, B, C…), are statistically different ($P<0.05$).

Table 6. Sensorial analysis measured in the smoked fillets of *D. labrax* during the 35 days of preservation, in the five smoking processes (I, II, III, IV, V).

		I	II	III	IV	V
**Day of storage**	**Type of brine solution**			**Odor**		
1	NaCl	4.00±0.00$^{ab/A}$	4.00±0.00$^{ab/A}$	4.00±0.00$^{ab/A}$	4.67±0.00$^{a/B}$	3.00±0.00$^{a/C}$
	NaCl/KCl	4.50±0.71$^{bc/A}$	4.00±0.00$^{ab/A}$	4.33±0.00$^{b/A}$	4.67±0.00$^{a/A}$	3.00±0.00$^{a/B}$
7	NaCl	5.00±0.00$^{c/A}$	4.50±0.00$^{ac/A}$	5.00±0.00$^{c/A}$	4.33±0.00$^{ab/A}$	5.00±0.00$^{c/A}$
	NaCl/KCl	4.75±0.35$^{c/A}$	3.75±0.35$^{b/B}$	5.00±0.00$^{c/A}$	4.00±0.00$^{abc/B}$	4.00±0.00$^{b/B}$
14	NaCl	4.00±0.00$^{ab/A}$	4.00±0.00$^{ab/A}$	3.00±0.00$^{d/B}$	3.75±0.35$^{bc/A}$	5.00±0.00$^{c/C}$
	NaCl/KCl	4.00±0.00$^{ab/A}$	3.50±0.71$^{b/B}$	3.00±0.00$^{d/C}$	3.00±0.00$^{d/C}$	4.00±0.00$^{b/A}$
21	NaCl	5.00±0.00$^{c/A}$	5.00±0.00$^{c/A}$	2.83±0.24$^{de/B}$	4.00±0.00$^{abc/C}$	4.00±0.00$^{b/C}$
	NaCl/KCl	3.00±0.00$^{de/A}$	5.00±0.00$^{c/B}$	2.50±0.71$^{e/C}$	4.00±0.00$^{abc/D}$	4.00±0.00$^{b/D}$
28	NaCl	3.00±0.00$^{de/A}$	4.17±0.24$^{ab/B}$	4.00±0.00$^{ab/B}$	4.00±0.00$^{abc/B}$	3.75±0.35$^{b/B}$
	NaCl/KCl	3.00±0.00$^{de/A}$	3.00±0.00$^{d/A}$	4.00±0.00$^{ab/B}$	4.00±0.00$^{abc/B}$	3.00±0.00$^{a/A}$
35	NaCl	3.50±0.71$^{ad/A}$	3.67±0.00$^{b/A}$	4.00±0.00$^{ab/A}$	4.00±0.00$^{abc/A}$	3.00±0.00$^{a/B}$
	NaCl/KCl	2.75±0.35$^{e/A}$	3.83±0.24$^{ab/B}$	3.50±0.71$^{a/AB}$	3.50±0.24$^{c/AB}$	3.00±0.00$^{a/AB}$
				**Colour**		
1	NaCl	4.00±0.00$^{a/A}$	4.00±0.00$^{ab/A}$	3.33±0.00$^{ab/B}$	4.83±0.24$^{a/C}$	3.00±0.00$^{a/B}$
	NaCl/KCl	4.00±0.00$^{a/A}$	4.00±0.00$^{ab/A}$	3.00±0.00$^{bc/B}$	4.67±0.00$^{ab/C}$	3.00±0.00$^{a/B}$
7	NaCl	4.00±0.00$^{a/A}$	4.00±0.00$^{ab/A}$	4.50±0.00$^{d/A}$	4.33±0.00$^{bc/A}$	4.00±0.00$^{b/A}$
	NaCl/KCl	4.00±0.00$^{a/A}$	4.00±0.00$^{ab/A}$	4.25±0.35$^{d/A}$	4.17±0.24$^{c/A}$	4.00±0.00$^{b/A}$
14	NaCl	4.00±0.00$^{a/A}$	3.50±0.71$^{ade/A}$	3.00±0.00$^{bc/B}$	4.00±0.00$^{c/A}$	3.75±0.35$^{b/A}$
	NaCl/KCl	4.00±0.00$^{a/A}$	3.25±0.35$^{de/B}$	3.00±0.00$^{bc/B}$	4.00±0.00$^{c/A}$	3.00±0.00$^{a/B}$
21	NaCl	3.00±0.00$^{b/A}$	4.50±0.71$^{b/B}$	2.33±0.00$^{ce/C}$	3.00±0.00$^{d/A}$	3.00±0.00$^{a/A}$

	NaCl/KCl	3.33±0.47$^{b/A}$	5.00±0.00$^{c/B}$	2.17±0.24$^{e/C}$	4.00±0.00$^{c/D}$	2.00±0.00$^{d/C}$
28	NaCl	3.00±0.00$^{b/A}$	4.00±0.00$^{ab/B}$	3.00±0.00$^{bc/A}$	4.00±0.00$^{c/B}$	2.00±0.00$^{d/C}$
	NaCl/KCl	3.00±0.00$^{b/A}$	3.00±0.00$^{d/A}$	3.00±0.00$^{bc/A}$	4.17±0.24$^{bc/B}$	2.50±0.71$^{c/A}$
35	NaCl	3.00±0.00$^{b/A}$	3.00±0.00$^{b/A}$	3.75±0.35$^{a/B}$	3.00±0.00$^{d/A}$	3.00±0.00$^{a/A}$
	NaCl/KCl	3.00±0.00$^{b/A}$	3.67±0.00$^{ab/B}$	3.50±0.71$^{ab/B}$	3.00±0.00$^{d/A}$	3.00±0.00$^{a/A}$
				**Appearance**		
1	NaCl	4.00±0.00$^{a/A}$	4.00±0.00$^{ab/A}$	3.17±0.24$^{a/B}$	4.67±0.00$^{a/C}$	3.00±0.00$^{ab/B}$
	NaCl/KCl	4.00±0.00$^{a/A}$	4.00±0.00$^{ab/A}$	3.00±0.00$^{a/B}$	4.67±0.00$^{a/C}$	4.00±0.00$^{c/A}$
7	NaCl	4.00±0.00$^{a/A}$	4.00±0.71$^{ab/A}$	4.50±0.00$^{b/A}$	4.33±0.00$^{ab/A}$	3.50±0.71^{bcB}
	NaCl/KCl	4.00±0.00$^{a/A}$	4.50±0.71$^{bc/A}$	4.25±0.35$^{b/A}$	3.83±1.18$^{ab/AB}$	3.75±0.35$^{c/B}$
14	NaCl	4.00±0.00$^{a/A}$	3.50±0.71$^{ab/AB}$	3.00±0.00$^{ac/B}$	4.00±0.00$^{ab/A}$	3.50±0.00$^{bc/AB}$
	NaCl/KCl	4.00±0.00$^{a/A}$	3.75±0.35$^{ab/A}$	3.50±0.71$^{a/AB}$	4.00±0.00$^{ab/A}$	3.00±0.00$^{ab/B}$
21	NaCl	4.00±0.00$^{a/A}$	4.50±0.71$^{bc/B}$	2.33±0.00$^{c/C}$	3.00±0.00$^{c/D}$	3.00±0.00$^{ab/D}$
	NaCl/KCl	3.17±0.24$^{bc/A}$	5.00±0.00$^{c/B}$	2.83±0.24$^{ac/A}$	4.00±0.00$^{ab/C}$	2.00±0.00$^{d/D}$
28	NaCl	3.25±0.35$^{bc/AB}$	3.67±0.00$^{ab/B}$	2.83±0.24$^{ac/A}$	3.50±0.71$^{bc/B}$	2.50±0.71$^{a/A}$
	NaCl/KCl	3.50±0.71$^{ab/A}$	3.50±0.71$^{ab/A}$	3.00±0.00$^{ac/A}$	4.17±0.24$^{ab/B}$	3.00±0.00$^{ab/A}$
35	NaCl	4.00±0.00$^{a/A}$	3.00±0.00$^{a/B}$	3.25±0.35$^{a/B}$	3.00±0.00$^{c/B}$	3.00±0.00$^{ab/B}$
	NaCl/KCl	3.00±0.00$^{c/A}$	3.50±0.24$^{ab/AB}$	3.50±0.71$^{a/AB}$	3.00±0.00$^{c/A}$	4.00±0.00$^{c/B}$
				**Taste**		
1	NaCl	5.00±0.00$^{a/A}$	4.00±0.00$^{a/B}$	3.67±0.00$^{ad/B}$	4.67±0.00$^{a/A}$	3.00±0.00$^{a/C}$
	NaCl/KCl	5.00±0.00$^{a/A}$	4.00±0.00$^{a/B}$	4.00±0.00$^{a/B}$	4.67±0.00$^{a/A}$	3.00±0.00$^{a/C}$
7	NaCl	5.00±0.00$^{a/A}$	5.00±0.00$^{b/A}$	4.50±0.00$^{b/AB}$	4.33±0.00$^{a/AB}$	4.00±0.00$^{b/B}$
	NaCl/KCl	3.75±1.06$^{b/A}$	4.00±0.00$^{a/A}$	5.00±0.00$^{c/B}$	4.33±0.47$^{a/AB}$	4.00±0.00$^{b/A}$
14	NaCl	4.00±0.00$^{b/A}$	4.00±0.00$^{a/A}$	3.00±0.00$^{de/B}$	4.50±0.71$^{a/C}$	4.00±0.00$^{b/A}$
	NaCl/KCl	4.00±0.00$^{b/A}$	3.75±0.35$^{a/A}$	3.00±0.00$^{de/B}$	4.00±0.00$^{a/A}$	4.00±0.00$^{b/A}$
21	NaCl	5.00±0.00$^{a/A}$	3.00±0.00$^{d/B}$	2.67±0.47$^{e/B}$	4.00±0.00$^{a/C}$	3.00±0.00$^{a/B}$
	NaCl/KCl	4.00±0.00$^{b/A}$	2.25±0.35$^{c/B}$	2.50±0.71$^{e/B}$	4.00±0.00$^{a/A}$	2.00±0.00$^{c/B}$
28	NaCl	3.00±0.00$^{c/A}$	4.00±0.00$^{a/B}$	3.00±0.00$^{de/A}$	4.00±0.00$^{a/B}$	2.00±0.00$^{c/C}$
	NaCl/KCl	3.00±0.00$^{c/A}$	3.00±0.00$^{d/A}$	3.00±0.00$^{de/A}$	4.00±0.00$^{a/B}$	3.00±0.00$^{a/A}$
35	NaCl	3.00±0.00$^{c/A}$	3.67±0.00$^{a/B}$	3.50±0.00$^{ad/B}$	3.00±0.00$^{b/A}$	3.00±0.00$^{a/A}$
	NaCl/KCl	3.00±0.00$^{c/A}$	3.83±0.24$^{a/B}$	3.50±0.71$^{ad/AB}$	3.00±0.00$^{b/A}$	3.00±0.00$^{a/A}$

Data within a column, showing different superscript letters (a, b, c…), are statistically different ($P<0.05$). Data within a row, showing different superscript letters (A, B, C…), are statistically different ($P<0.05$).
